# Clinical outcomes after reirradiation with dose painting of recurrent or second primary head and neck cancer

**DOI:** 10.3389/fonc.2026.1966123

**Published:** 2026-09-30

**Authors:** Einar Dale, Morten E. Evensen, Cecilie D. Amdal, Torbjørn Furre, Jon M. Moan, Liv Cathrine Heggebø, Ayca M. Løndalen, Eirik Malinen

**Affiliations:** 1 Department of Oncology, Oslo University Hospital, Oslo, Norway; 2 Section of Oncology, Drammen Hospital, Vestre Viken Hospital Trust, Drammen, Norway; 3 Department of Medical Physics, Oslo University Hospital, Oslo, Norway; 4 Department of Nuclear Medicine, Innlandet Hospital Trust, Gjøvik, Norway; 5 Department of Radiation Biology, Institute for Cancer Research, Oslo University Hospital, Oslo, Norway; 6 Department of Physics, University of Oslo, Oslo, Norway

**Keywords:** dose painting, FDG-PET/CT, head and neck cancer, radiotherapy, reirradiation

## Abstract

**Purpose:**

Recurrent or second primary head and neck cancer (rsHNC) presents a radiotherapeutic challenge due to limited tolerance of previously irradiated tissues. These patients could benefit from dose painting (DP), which focuses on delivering maximal dose to areas of high tumor burden while respecting dose constraints to organs at risk. In this study, we compare clinical outcomes in one group receiving standard reirradiation with a group receiving 18F-fluorodeoxyglucose (FDG) PET/CT-guided DP.

**Methods:**

Our standard reirradiation regimen is hyperfractionated radiotherapy (RT) of 1.5 Gy x 40 (60 Gy total dose). For DP, we defined two FDG-based prescription volumes receiving at least 65 Gy and 70 Gy. Thirteen patients receiving standard RT were compared with 10 DP patients. Toxicity was scored using CTCAE v3.0. Disease-related endpoints were locoregional control (LRC), disease-free survival (DFS) and overall survival (OS).

**Results:**

The mean composite toxicity score was maximal at the end of RT, with varying patterns across the CTCAE items. No significant difference in toxicity was found between the groups except for dysphagia at 6 months, where the standard RT group had worse scores (p=0.01). No differences were found in LRC and DFS. Two-year OS was 50% in the DP group versus 15% in the standard group (p=0.04), although this may reflect higher comorbidity in the standard group.

**Conclusion:**

DP of rsHNC did not increase toxicity compared with standard reirradiation. No evidence of improved disease control was found. However, DP may benefit selected patients, and future studies should focus on identifying these candidates.

**Clinical trial registration:**

## Introduction

Thirty to fifty percent of patients with locally advanced head and neck cancer (HNC) develop recurrence after primary treatment, which commonly includes radiotherapy (RT) (1). Many recurrences are unresectable, or patients are not candidates for further surgery. Outcomes remain poor, with fewer than 20% of patients with locoregional relapse surviving beyond 5 years (2). Second primary HNC represents a related clinical challenge, although prognosis is more favorable than for recurrent disease (3, 4).

Reirradiation at high dose is challenging because previously irradiated normal tissues have limited residual tolerance, increasing the risk of severe acute and late toxicity. Advances in RT delivery, including volumetric-modulated arc therapy (VMAT), allow improved dose conformity and reduction of high-dose exposure to surrounding tissues. For recurrent and second primary HNC (rsHNC), these approaches have been associated with overall survival (OS) of around 30%, at the cost of increased late morbidity (5).

Most local HNC recurrences arise within the original gross tumor volume (GTV), particularly in subregions with high 18F-fluorodeoxyglucose (FDG) uptake on positron emission tomography/computed tomography (PET/CT) images (6–8). Dose painting (DP) may be used to escalate the dose to hypermetabolic regions within the recurrent tumor, based on the premise that FDG uptake reflects aggressive tumor biology. DP may be delivered by VMAT, which will normally limit exposure to organs at risk. FDG-PET-guided DP may be applicable both for primary and recurrent HNC.

Clinical evidence for DP in the reirradiation setting remains limited. To our knowledge, in addition to our previous work (9), only two clinical studies have been reported (10, 11) warranting further investigations. In the current study, we aimed to assess the feasibility of FDG-PET-guided DP in patients with rsHNC and compare the clinical outcome with a group of patients receiving standard reirradiation in our institution.

## Materials and methods

### Patients

This sub-study is part of a larger prospective, longitudinal observational cohort study titled “RIOT - Reirradiation or Other Treatment of patients with Head and Neck cancer unsuited for radical RT, evaluation with patient reported outcomes (RIOT)” conducted at Oslo University Hospital (12). Inclusion criteria for the sub-study were histologically or cytologically confirmed squamous cell carcinoma in the head and neck region, planned hyperfractionated reirradiation (HFR) without concomitant systemic therapy, age ≥18 years, with ability to respond to questionnaires. The exclusion criterion was previous reirradiation. Reirradiation was not recommended within 6 months after primary RT and for patients with RT-related severe trismus, ulcerations or osteoradionecrosis. In the present sub-study, patients with inoperable disease planned for HFR were included and consisted of two groups: 1) A control group of 13 patients who received standard HFR (no DP). 2) Ten patients who received FDG-PET-guided DP (ClinicalTrials.gov identifier NCT02921581). The primary endpoint was feasibility (success rate and workload) of implementing the DP technique. Secondary endpoints were toxicity and tumor response.

This study was approved by the Regional Ethics Committee (Regional Committees for Medical and Health Research Ethics, Southeast Norway). Informed consent was obtained from all patients.

### Treatment planning

All imaging for treatment planning was performed on an RT-compatible flat table with head support and a radiotherapy fixation mask. The patients in the control group underwent a contrast-enhanced CT scan (Brilliance Big Bore CT, Philips Healthcare, Best, The Netherlands) from the skull base to mid chest with the arms down. The patients in the DP group had a contrast-enhanced FDG-PET/CT scan (Biograph 32, Siemens Healthcare, Munich, Germany). Treatment planning was performed using Raystation (v. 4.5/5.0, RaySearch Laboratories, Stockholm, Sweden).

Our standard reirradiation regimen consisted of 1.5 Gy twice daily to 60 Gy over 4 weeks. The GTV (defined as the macroscopic disease visible on CT) was expanded by either (i) 5 mm to generate the clinical target volume (CTV, accounting for microscopic disease) or (ii) 10 mm margin in which also limited elective lymph node areas were included. In the latter case, the CTV received a dose of 45 Gy over the first 3 weeks, followed by an additional boost of 15 Gy to the GTV. In the former case, the entire CTV received a dose of 60 Gy. Standard reirradiation was given with a homogenous dose to the CTV. Planning treatment volume margin was 3 mm.

Our DP method has been described previously (9). In brief, two prescription volumes (PV33 and PV66) were identified from the PET-images using a linear dose prescription formula converting FDG uptake to dose (13). The span of FDG uptake in the tumor was divided into three. PV33 encompassed 2/3 of the intermediate to high intensities while PV66 encompassed the 1/3 highest intensities. PV33 and PV66 received 65 Gy and 70 Gy, respectively, with a maximum point dose of 75 Gy. DP was given the first 2 out of 4 weeks treatment with a dose per fraction to PV33 and PV66 of 1.75 Gy and 2.0 Gy (Dmax 2.25 Gy), respectively. During the last 2 weeks of the treatment, the CTV received a homogeneous dose per fraction of 1.5 Gy.

Dose calculation was done by Raysearch’s implementation of collapsed cone v. 3.0/3.2 with a grid size of 2 mm. The plans were checked for dosimetric accuracy using an Arc Check phantom (Sun Nuclear Corporation, Melbourne, FL, USA). All plans were within the tolerance limit used at our hospital with a gamma pass rate above 95% for the 3%/3 mm criteria. Treatment was given on a Varian TrueBeam STx (Varian Medical Systems, Palo Alto, CA, USA) with a HD120 MLC (2.5 mm and 5 mm central and outer leaf thickness, respectively) and 6 MV photons.

### Follow-up

Toxicity (Common Terminology Criteria for Adverse Events, CTCAE v3.0) was scored for 13 (mucositis, dysphagia, taste, edema, hoarseness, dermatitis, xerostomia, fistula, trismus, osteoradionecrosis, fibrosis, bleeding, local pain) at baseline, at the end of RT, and 3, 6 and 12 months after baseline. Furthermore, a composite score; the mean value of all 13 scores was calculated. Disease-related endpoints were loco-regional control (LRC), disease-free survival (DFS) and OS. For the DP group, follow-up FDG-PET/CT was performed 5.5 months after inclusion (4 months after reirradiation), thereafter as clinically indicated. For the control group no per protocol imaging was specified but the institutional practice was to perform CT 3 months after RT and then as clinically indicated.

### Statistics

Frequencies in contingency tables were compared using Fisher’s exact test. The Mann–Whitney U test was used for group comparisons. Local, regional, and distant failure were determined by biopsy when available, or by radiological assessment if biopsy was not warranted. For calculation of survival, the Kaplan–Meier method was used. Time to event was measured from inclusion. For LRC, either local or regional failure was counted as an event; for DFS, distant failure and death were additionally counted. End of follow-up was December 24, 2025.

P-values <0.05 (two-sided) were considered statistically significant. The data were analyzed using RStudio 2023.06.2 + 561 (14).

## Results

### Patients and treatment

Ten patients with rsHNC were enrolled and successfully reirradiated with DP between 2016-2020. DP introduced additional steps in the treatment planning workflow, increasing planning time by approximately 1–2 hours compared with standard radiotherapy. In addition, approximately 30 minutes were required for treatment approval and pre-treatment quality assurance prior to treatment initiation. Treatment delivery was uncomplicated, and no additional medical care was required during treatment. **Figure 1** shows an example of planning FDG-PET/CT, DP plan and imaging 4 months after reirradiation.

**Figure 1:**
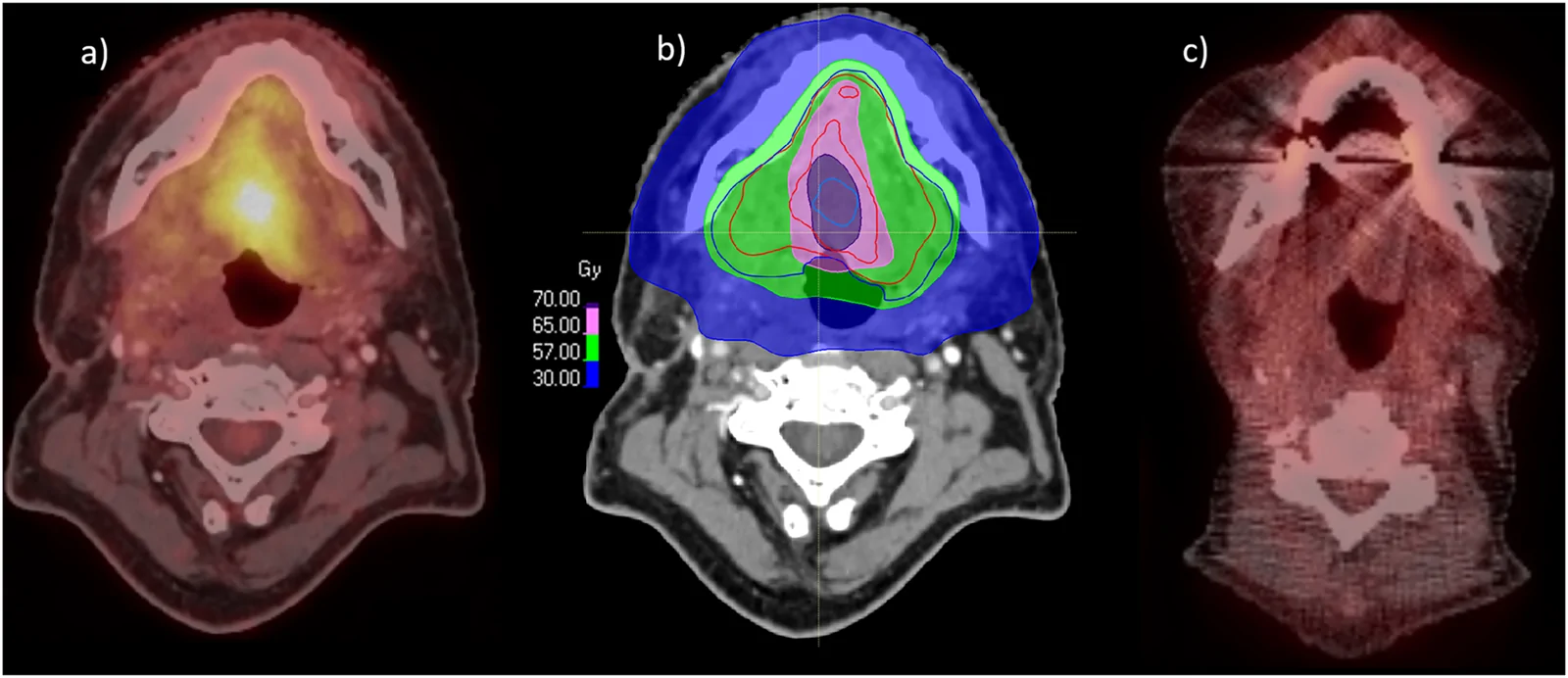
This patient received radiotherapy (RT) for HPV positive oropharyngeal cancer (right side) 7 years before current diagnosis of second primary in the tongue; **(a)** planning FDG-PET/CT, **(b)** dose painting plan: 57 Gy to the CTV (outer blue contour) Pink shade: 65 Gy to PV33. Purple shade: 70 Gy to PV66. **(c)** FDG-PET/CT with no FDG uptake 4 months after dose painting. FDG-PET/CT with no FDG uptake 4 months after dose painting.

The standard treatment group consisted of 13 patients receiving reirradiation with a homogeneous dose to the CTV in the period 2015-2018. In this group, there were more patients with second primary cancer compared to the DP group, but this difference was not statistically significant (p = 0.21; **Table 1**). In the DP group there were more patients with human papilloma virus related oropharynx cancer compared to the standard group (p = 0.001). Compared to the DP group, there was a tendency towards poorer performance status, more comorbidity, more smoking, use of alcohol, more weight loss and increased baseline toxicity scores prior to treatment in the standard group.

**Table 1:** Baseline characteristics.

Characteristic	Category / measure	Standard radiotherapy N = 13	Dose painting N=10
Age (years)	Mean (range)	67.9 (50, 89)	64.8 (38, 82)
	Median	68	65.5
Gender n (%)	Male	10 (77)	9 (90)
	Female	3 (23)	1 (10)
Disease status n (%)	Relapse/progression	5 (38)	7 (70)
	Second primary	8 (62)	3 (30
Tumor location n (%)	Oral cavity	3 (23)	1 (10)
	Oropharynx	4* (31)	8** (80)
	Hypopharynx	4 (31)	0 (0)
	Larynx	2 (15)	0 (0)
	Unknown primary	0 (0)	1 (10)
WHO performance status n (%)	0	1 (8)	4 (40)
	1	7 (54)	5 (50)
	2	5 (38)	1 (10)
	3-4	0 (0)	0 (0)
Charlson Co-morbidity score n (%)	0	4 (31)	7 (70)
	1	4 (31)	2 (20)
	≥ 2	5 (38)	1 (10)
Weight loss last six months n (%)	≤ 5%	3 (23)	9 (90)
	> 5%	9 (69)	1 (10)
	Unknown	1 (8)	0 (0)
Smoking history n (%)	Never	3 (23)	1 (10)
	Stopped	5 (38)	4 (40)
	Ongoing	5 (38)	5 (50)
	< 20 pack years	4 (31)	4 (40)
	≥ 20 pack years	6 (46)	4 (40)
	Unknown pack years	3 (23)	2 (20)
Alcohol history n (%)	Never or occasionally	3 (23)	7 (70)
	Weekly	5 (38)	2 (20)
	Daily	5 (38)	1 (10)
Nutritional support n (%)	No	9 (69)	9 (90)
	Nasogastric tube	1 (8)	0 (0)
	Percutaneous endoscopic gastrostomy	3 (23)	1 (10)
Opioid (regular or on demand) n (%)	No	8 (62)	6 (60)
	Yes	5 (38)	4 (40)

*3 HPV-unrelated, 1 unknown HPV status.

**7 HPV-related (including cancer of unknown primary) and 2 HPV-unrelated.

The mean time since primary RT was longer in the standard group compared to the DP group; 124 months vs 52 months (p = 0.04). Otherwise, treatment characteristics were similar (**Table 2**).

**Table 2:** Treatment characteristics, mean (minimum, maximum).

Characteristic	Standard radiotherapy	Dose painting
Time since first radiotherapy (months)	124 (18, 328)	52 (13, 100)
Duration of reirradiation (days)	27 (25, 30)	29 (25, 36)
GTV (cc)	19.4 (2.0, 65.6)	18.7 (5.5, 30.9)

In the DP group, the mean PV33 and PV66 were quite small; 6.3 cc and 1.7 cc, respectively, compared to a mean GTV of 18.7 cc. Adequate target coverage was obtained for all 10 patients receiving DP, with 95% of the prescribed dose covering each of the CTV, PV33 and PV66.

### Toxicity

There were no statistically significant differences between the DP and standard groups in the proportion of patients experiencing any grade 3 toxicity at any of the time points (**Table 3**). In **Figure 2**, the median CTCAE score for xerostomia and dysphagia and the mean composite score of all 13 CTCAE scores are shown. The CTCAE scores were highest at the end of RT, with some variation between specific toxicities. Comparing the two groups at each time point, no difference in toxicity was found except for worse dysphagia at 6 months in the standard group (p=0.01). Three patients in the standard group died of carotid blowout at 2, 5 and 8 months, whereas no such events occurred in the DP group.

**Table 3:** Acute and late toxicity.

CTCAE term	Pre- treatment		End of treatment		3 months		6 months		12 months		36 months	
	Standard RT	Dose painting	Standard RT	Dose painting	Standard RT	Dose painting	Standard RT	Dose painting	Standard RT	Dose painting	Standard RT	Dose painting
	n = 13 n (%)	n = 10 n (%)	n = 13 n (%)	n = 10 n (%)	n = 12 n (%)	n = 10 n (%)	n = 7 n (%)	n = 9 n (%)	n = 2 n (%)	n = 8 n (%)	n = 2 n (%)	n = 4 n (%)
Mucositis	0 (0)	0 (0)	0 (0)	2 (0)	0 (0)	1 (10)	0 (0)	0 (0)	0 (0)	0 (0)	0 (0)	0 (0)
Dysphagia	4 (31)	0 (0)	7 (54)	4 (40)	5 (45)	2 (20)	3 (38)	0(0)	2 (100)	1 (12)	2 (100)	0 (0)
Taste alteration	0 (0)	0 (0)	0 (0)	0 (0)	0 (0)	0 (0)	0 (0)	0 (0)	0 (0)	0 (0)	0 (0)	0 (0)
Edema	0 (0)	0 (0)	0 (0)	0 (0)	0 (0)	0 (0)	0 (0)	0 (0)	0 (0)	0 (0)	0 (0)	0 (0)
Hoarseness	0 (0)	0 (0)	1 (8)	0 (0)	1 (9)	1 (10)	0 (0)	0 (0)	1 (50)	0 (0)	1 (50)	0 (0)
Dermatitis	0 (0)	0 (0)	0 (0)	0 (0)	0 (0)	0 (0)	0 (0)	0 (0)	0 (0)	0 (0)	0 (0)	0 (0)
Xerostomia	2 (15)	0 (0)	2 (15)	2 (20)	1 (9)	2 (20)	1 (12)	0 (0)	1 (50)	0 (0)	0 (0)	0 (0)
Fistula	0 (0)	0 (0)	0 (0)	0 (0)	0 (0)	0 (0)	0 (0)	0 (0)	0 (0)	0 (0)	0 (0)	0 (0)
Trismus	1 (8)	0 (0)	2 (15)	2 (20)	0 (0)	0 (0)	0 (0)	1 (11)	0 (0)	1 (12)	0 (0)	1 (25)
Osteonecrosis	0 (0)	0 (0)	0 (0)	0 (0)	0 (0)	0 (0)	1 (12)	1 (11)	0 (0)	1 (12)	0 (0)	0 (0)
Indurations/ Fibrosis	0 (0)	0 (0)	1 (8)	1 (10)	2 (18)	1 (10)	3 (38)	0 (0)	1 (50)	1 (12)	1 (50)	1 (25)
Bleeding	0 (0)	0 (0)	0 (0)	0 (0)	0 (0)	0 (0)	0 (0)	0 (0)	0 (0)	0 (0)	0 (0)	0 (0)
Pain (local)	1 (8)	1 (10)	2 (15)	1 (10)	1 (9)	0 (0)	1 (12)	0 (0)	0 (0)	1 (12)	0 (0)	0 (0)
Any toxicity	5 (38)	1 (10)	8 (62)	5 (50)	7 (58)	4 (40)	5 (71)	2 (22)	2 (100)	4 (50)	2 (100)	1 (25)

Number of patients (%) with grade ≥ 3 CTCAE. Bleeding: Three patients in the standard group died of carotid blowout at 2, 5 and 8 months. These patients were not scored grade ≥ 3 at scheduled follow-up visits. Time was measured from inclusion, approximately 1 week before start of radiotherapy (RT).

**Figure 2:**
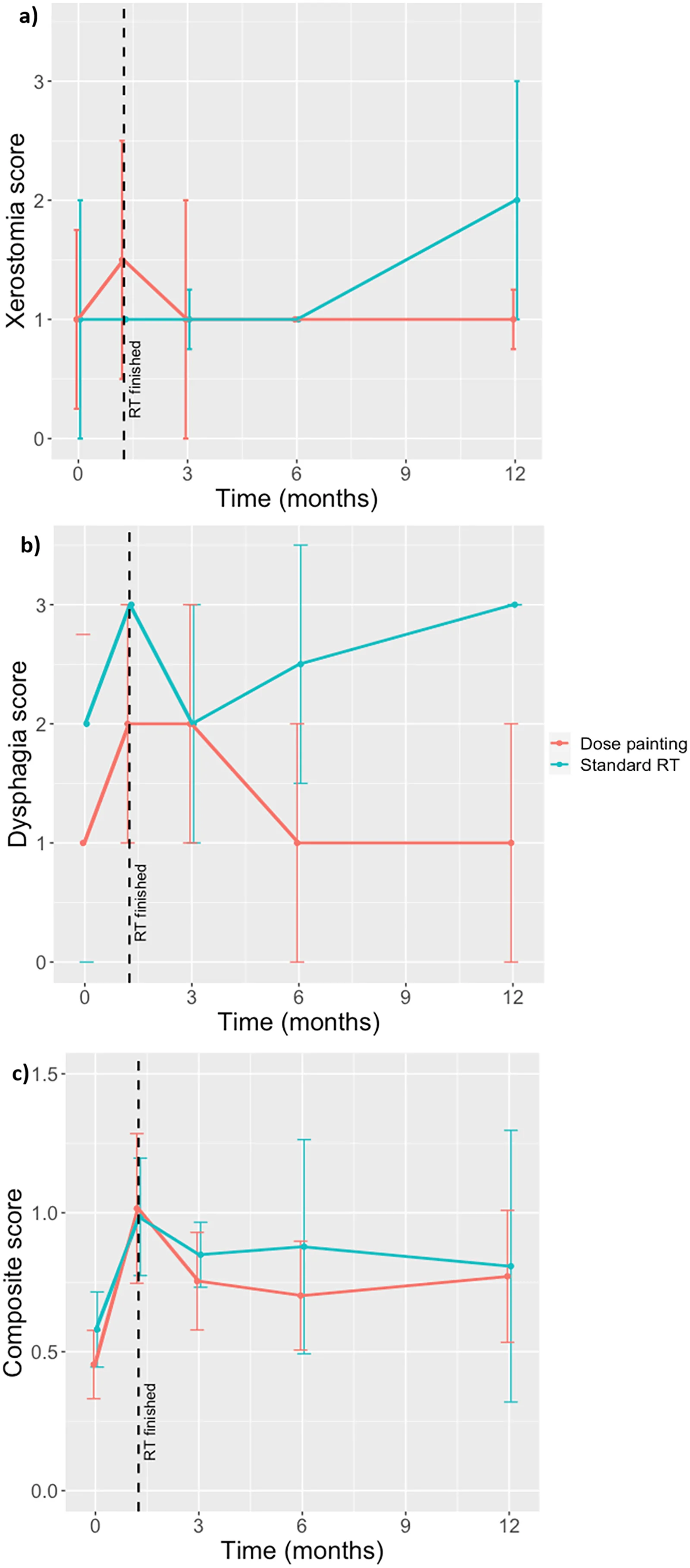
CTCAE toxicity scores. **(a)** Median xerostomia score and interquartile range (IQR). **(b)** Median dysphagia score and IQR. **(c)** Overall mean of 13 scores and 95% confidence intervals.

### Survival

The 2-year LRC was 22% (95% CI; 7%, 76%) and 45% (23%, 89%) in the DP group and the standard group, respectively. The 2-year DFS was 20% (6%, 69%) and 15% (4%, 55%). The 2-year OS was 50% (27%, 93%) and 15% (4%, 55%), and survival was significantly higher in the DP group (p=0.04; **Figure 3**).

**Figure 3:**
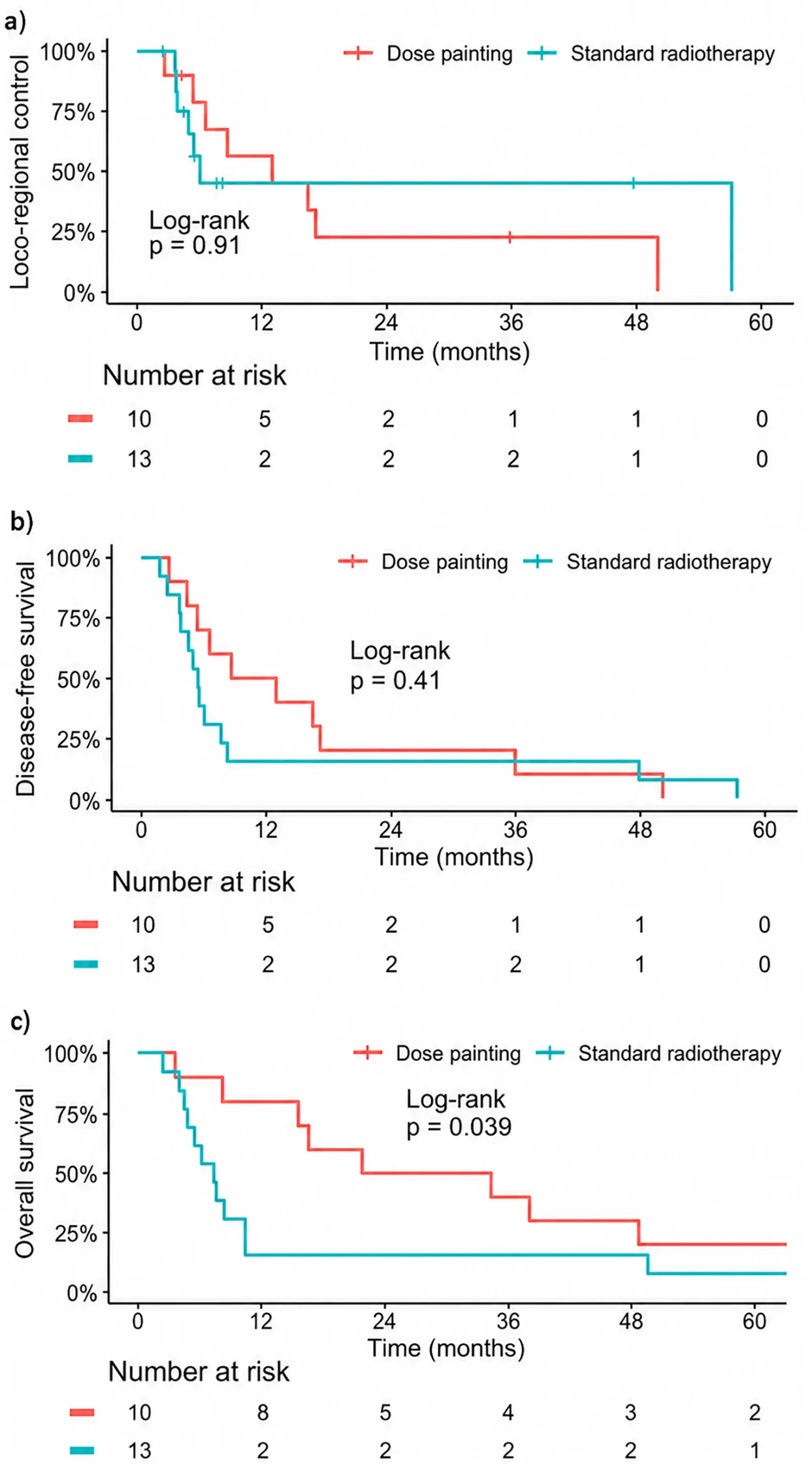
**(a)** Loco-regional control. **(b)** Disease-free survival. **(c)** Overall survival.

There were seven loco-regional recurrences in the DP group. Six of these were inside the high-dose area (PTV) and for two of these there were also recurrences outside the PTV. There were six loco-regional recurrences in the standard group. Four of these were inside the PTV.

In the DP group, eight out of ten patients died of progression/recurrence. Six of these received palliative systemic therapy. One patient with a contralateral regional recurrence survived after neck surgery followed by a further course of radiotherapy. One patient died 46 months after RT with no evidence of disease 5 months before death (last follow-up). In the standard group, 7 out of 13 patients died of cancer progression/recurrence, five of these received systemic therapy and two patients had tumor causing carotid blow out. Of the remaining six patients without evidence of disease, one died of carotid blow out, three died of infection, one died of myocardial infarction, and one died of esophageal cancer.

## Discussion

Dose painting in the reirradiation setting requires integration of PET/CT imaging, heterogeneous dose prescription, treatment planning, and patient-specific quality assurance while maintaining safe exposure of previously irradiated normal tissues. In this study, FDG-PET-guided DP was delivered successfully to all patients, meeting the prespecified feasibility endpoint. The additional planning and pre-treatment quality-assurance procedures were manageable and did not prevent treatment delivery. These findings show that DP can be incorporated into a clinical reirradiation workflow and support its further prospective evaluation.

To improve the understanding of the clinical outcomes in the DP group, we included a control group of 13 patients from a larger observational cohort study. The patients in the control group had also received high-dose reirradiation for inoperable disease but with homogeneous target dose. The patients in the control group had poorer performance status and more comorbidity. After reirradiation, the acute and late toxicity in the two groups were comparable, lending support to the hypothesis that DP does not increase toxicity for the dose levels used in this study.

Notably, fatal bleeding (carotid blow out) was the most serious late event, occurring in three patients in the control group, one of whom had no documented cancer progression at the time of the event.

We have found two other DP reirradiation studies in the literature (10, 11). Schatteman et al. did a pilot study (n=10) similar to the current investigation, including rsHNC. The dose prescribed to the target volume was 60.0 - 100.5 Gy EQD2Gy (alfa/beta = 3 Gy) compared to 54.0-75.8 EQD2Gy in the present study. Concomitant cetuximab was given to six of the patients in the Schatteman study, and no control group was included. They reported similar toxicity rates as in our study, despite using a different late toxicity grading system (SOMA-LENT). Tan et al. presented a randomized reirradiation trial (n=150) on MRI-guided DP for patients with nasopharyngeal carcinoma using a target dose of 60–68 Gy EQD2Gy compared to homogeneous dose of 60 EQD2Gy in the control group (11). The patients received induction chemotherapy with three cycles of gemcitabine and cisplatin. They reported eight patients with massive nasal bleeding and six of these died. There were no differences in grade 3 or higher toxicity.

We did not find evidence of increased treatment effect in the DP group apart from increased OS, which we attribute to less comorbidity in the DP group. The 2-year LRC and OS observed in the present study were comparable to those reported by Langendijk et al, with 2-year LRC and OS rates of 27% and 28%, respectively (15). The study of Schatteman et al. (10) also found similar disease control rates as in the present study. In the randomized trial by Tan et al. (11) there was evidence of better disease outcome in the DP group, but this has less relevance for non-nasopharyngeal HNC as in our study.

In the present study, the 2-year LRC was numerically lower in the DP group than in the standard group (22% vs. 45%), although this difference was not statistically significant. The 2-year LRC estimates should be interpreted with caution, as they are based on very few patients remaining at risk at this time point. Nevertheless, we chose to report the 2-year estimates to facilitate comparison with the results reported by Langendijk et al. (15).

Most locoregional failures in the DP group occurred within the high-dose treatment volume. While this finding should be interpreted cautiously for such a small cohort, the dose escalation used in the present study may therefore have been insufficient to achieve control of aggressive recurrent disease. The two other reirradiation DP studies support further exploration of more intensive focal escalation (10, 11). However, potential gains in local control must be balanced against the narrow therapeutic window in previously irradiated tissues, where higher doses may increase the risk of severe late toxicity. Future approaches may therefore consider higher doses to smaller, anatomically confined subvolumes, with rigorous assessment of cumulative organ-at-risk doses and patient selection. Advanced optimization strategies such as multicriteria optimization (MCO) could potentially support such individualized planning by facilitating exploration of trade-offs between focal dose escalation and cumulative dose to previously irradiated organs at risk. However, while MCO has demonstrated dosimetric and workflow benefits in HNC planning (16), its clinical value specifically for reirradiation and DP remains to be established. Regarding patient selection, suitable candidates may be those with limited disease containing a well-defined FDG-avid subvolume that can be escalated without unacceptable cumulative dose to critical normal tissues. Favorable anatomy, including separation from the carotid arteries and other vulnerable structures may be particularly important. Clinical factors such as good performance status, limited comorbidity, a longer interval since previous radiotherapy, and absence of ulceration or vascular involvement may further support treatment selection.

In conclusion, DP for rsHNC was feasible, although workload was moderately increased. DP did not increase toxicity compared with conventional reirradiation, but we found no evidence of improved disease control. Still, DP is expected to give a benefit in selected patients, and a challenge in future studies is to identify these cases.

## Data Availability

The raw data supporting the conclusions of this article will be made available by the authors, without undue reservation.
